# Sterile inflammation and the NLRP3 inflammasome in cardiometabolic disease

**DOI:** 10.1016/j.bj.2023.100624

**Published:** 2023-06-17

**Authors:** Sungji Cho, Fan Ying, Gary Sweeney

**Affiliations:** Department of Biology, York University, Toronto, Ontario, Canada

**Keywords:** Sterile inflammation, NLRP3 inflammasome, Cardiometabolic diseases

## Abstract

Inflammation plays an important role in the pathophysiology of cardiometabolic diseases. Sterile inflammation, a non-infectious and damage-associated molecular pattern (DAMP)-induced innate response, is now well-established to be closely associated with development and progression of cardiometabolic diseases. The NOD-like receptor (NLR) family pyrin domain-containing 3 (NLRP3) inflammasome is well-established as a major player in sterile inflammatory responses. It is a multimeric cytosolic protein complex which regulates the activation of caspase-1 and subsequently promotes cleavage and release of interleukin (IL)-1 family cytokines, which have a deleterious impact on the development of cardiometabolic diseases. Therefore, targeting NLRP3 itself or the downstream consequences of NLRP3 activation represent excellent potential therapeutic targets in inflammatory cardiometabolic diseases. Here, we review our current understanding of the role which NLRP3 inflammasome regulation plays in cardiometabolic diseases such as obesity, diabetes, non-alcoholic steatohepatitis (NASH), atherosclerosis, ischemic heart disease and cardiomyopathy. Finally, we highlight the potential of targeting NLPR3 or related signaling molecules as a therapeutic approach.

Adequate onset and resolution of inflammation is a fundamental process in tissue maintenance and repair [1]. This involves intracellular, intercellular and interorgan communication with multiple complex regulatory mechanisms. Prolonged inflammation due to excess induction or inadequate resolution has been established as a contributor to the development and progression of cardiometabolic diseases, such as diabetes, atherosclerosis, cardiomyopathy and myocardial infarction [1]. Indeed, patients with elevated levels of systemic inflammatory markers also have a greater incidence of cardiometabolic diseases compared to the general population [2]. Therefore, targeting inflammation may offer a novel approach to reducing risk for those events.

The innate immune system serves as the initial line of defense against harmful circumstances caused by either pathogen-derived molecules or host-derived molecules. Innate immune cells play an important role in orchestrating the initial defense response of the host, relying on pattern recognition receptors (PRRs) to identify specific entities on the surface of pathogens and damaged host cells, as well as factors released by host cells [3]. Inflammatory triggers can be categorized into two types based on their origin: pathogen-associated molecular patterns (PAMPs) and damage-associated molecular patterns (DAMPs). Despite numerous shared mechanisms between them, it is important to note that only DAMP-induced inflammation is considered sterile [3].

The NOD-like receptor (NLR) family pyrin domain-containing 3 (NLRP3) is the most well-studied inflammasome and can be regulated by diverse stimuli to subsequently mediate the activation of caspase-dependent inflammatory responses [3]. Upon the activation by PAMPs or DAMPs-associated stimulators, NLRP3 recruits and activates caspase-1 with assistance of the adaptor unit ASC, which acts by bridging NLRP3 with pro-caspase-1 via PYD homophilic interaction, and subsequently processes targets such as pro-interleukin (IL) 1β and pro-IL-18 into biologically active forms of these cytokines that mediate the inflammatory responses [3]. The downstream consequences of NLRP3 inflammasome activation mediated via cytokines such as IL-1β and IL-18, have a deleterious impact on cardiometabolic diseases [3]. This review focuses on introducing the role of sterile inflammation in cardiometabolic diseases before delving into a specific focus on the NLRP3 inflammasome.

## Sterile inflammation: non-microbial inflammation

**Fig. 1:**
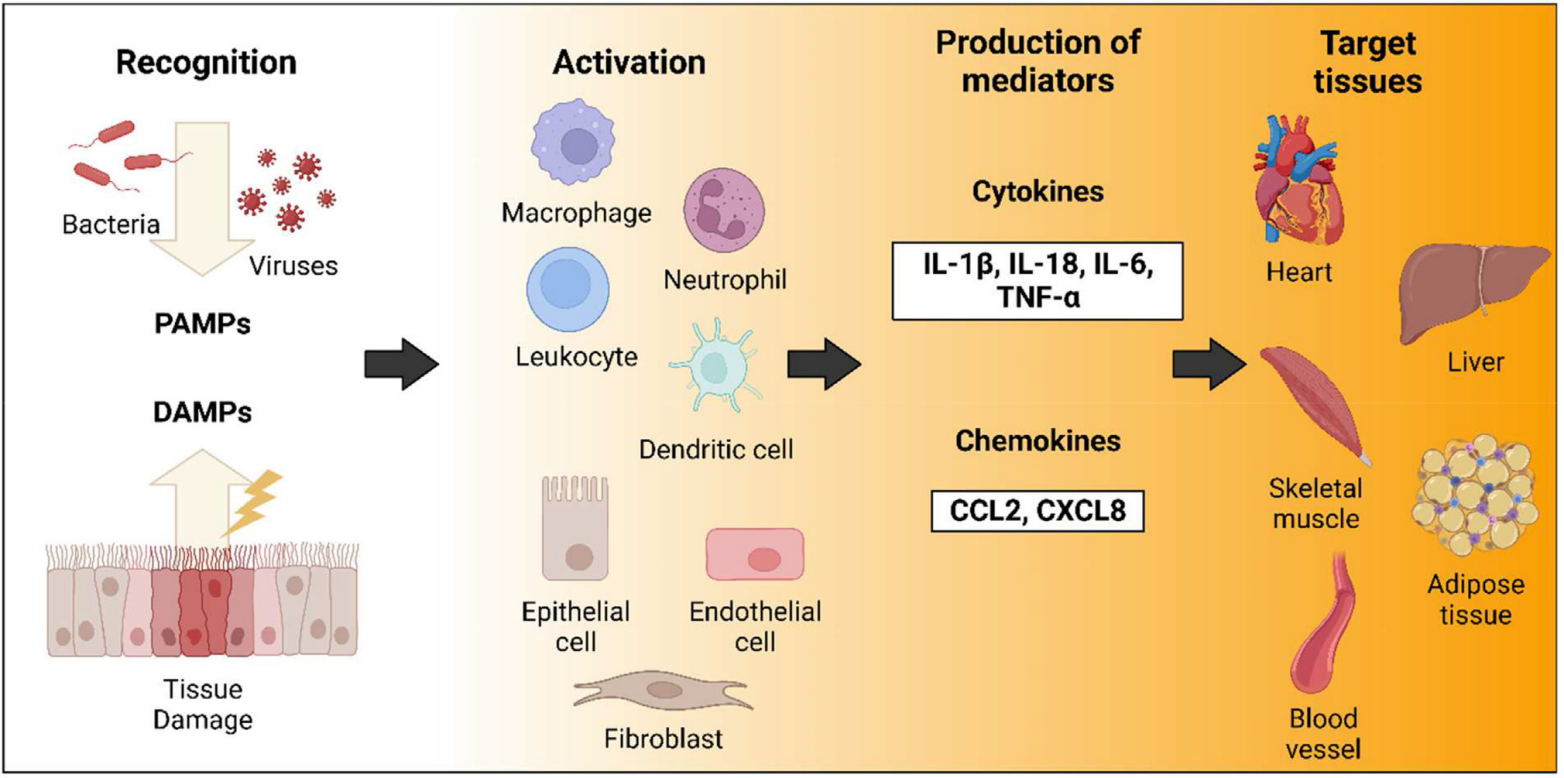
**Overview of mechanisms involved in initiating inflammation**. The overall execution of initiating inflammation consists of four steps: recognizing inflammatory triggers, activating the innate immune system, producing inflammatory mediators and effects of these mediators on target tissues. Upon responding to PAMPs and DAMPs, various innate immune cells such as macrophages, neutrophils, leukocytes and dendritic cells produce additional cytokines (such as IL-1β, IL-18, IL-6, TNF-α) and chemokines (CCL2, CXCL8) that lead to inflammation in target tissues such as heart, liver, skeletal muscle, adipose tissue, and blood vessels).

Inflammation is an essential biological reaction in response to the disruption of tissue homeostasis [1]. Classic innate immune function occurs upon encountering indicators of microorganism infection, such as bacteria or virus. Sentinel cells of the innate immune system, including macrophages, dendritic cells and mast cells, promptly initiate responses that further trigger the induction of inflammation. These responses involve eliminating the infectious agents and generating additional signaling mediators, such as cytokines and chemokines, which ultimately manifest in an appropriate inflammatory response [1,4]. Thus, the major function of classic innate inflammation is for eradication of infection and thus restoring tissue homeostasis. Unlike microbial inflammation, sterile inflammation refers to an inflammatory response that occurs without infection. It is not triggered by extracellular noxious stimuli, but primarily by factors released from damaged or necrotic cells [4]. Sterile inflammation is also known as DAMP-induced inflammation, which is mediated by the activation of specific receptors [4]. Substantial evidence has shown that DAMPs-induced inflammation is associated with various cardiometabolic diseases, including diabetes, nonalcoholic fatty liver diseases (NAFLD), ischemia-reperfusion injury, atherosclerosis and cardiac pressure overload [5]. Therefore, understanding the mechanisms of sterile inflammation and its resolution is essential to develop inflammation-targeted therapies against above diseases (Fig. 1).

## Mechanisms regulating onset and resolution of inflammation

During inflammation, collateral damage caused by mechanisms such as excess reactive oxygen species (ROS) from activated neutrophils is frequently observed. If this is prolonged, the trade-off between a favorable damage control response and unwanted tissue injury is perturbed [4]. Hence, in order to maintain health of the host, precise regulation of onset and resolution of inflammatory responses is critical [4].

**Fig. 2:**
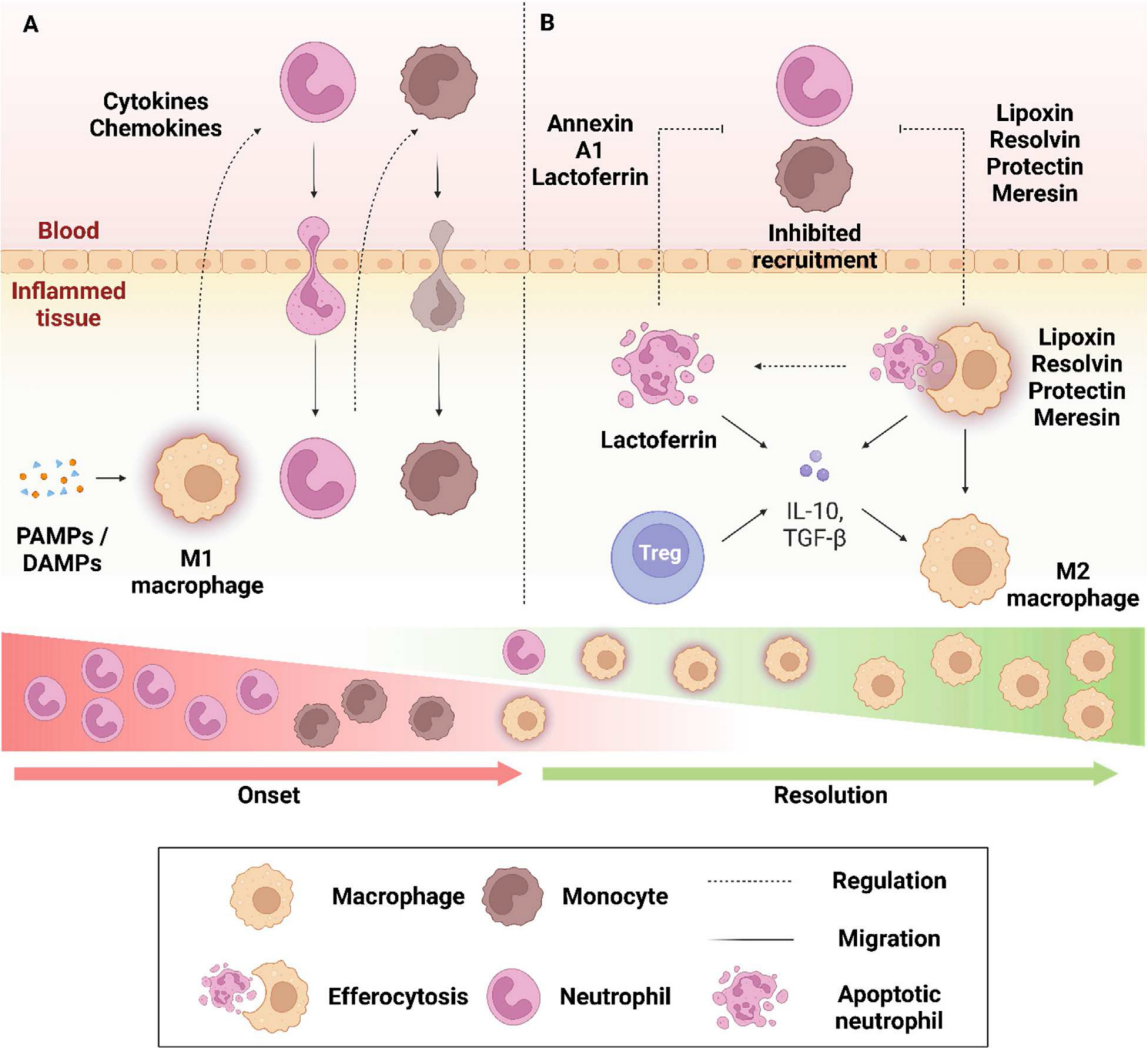
**The interplay of immune cells during onset & resolution of inflammation**. The inflammatory response can be divided into two phases: onset and resolution. Release of stimuli such as PAMPs and DAMPs from injured tissues initiates the inflammatory response by promoting the recruitment of neutrophils and monocytes, such as via chemokines secreted from M1 pro-inflammatory macrophages. During resolution, the recruitment of neutrophils and monocytes is inhibited and their apoptosis is induced, followed by macrophage polarization towards to M2 anti-inflammatory phenotype and infiltration of Tregs. Collectively, these contribute to repairing tissue damage and rebalancing immune homeostasis.

The original damage or injury is initially detected by tissue-resident innate immune cells, which are akin to an alarm that produces additional inflammatory mediators, including cytokines and chemokines to further propagate the immune response [6]. The most immediate effect of these mediators is to induce the recruitment of circulating neutrophils; one of the first cellular mediators of sterile inflammation that propagate early stages of the inflammatory response [6]. Once the neutrophils become activated when they migrate into the inflamed tissue, the activated neutrophils produce and release factors like ROS and reactive nitrogen species (RNS). However, if excessive or prolonged, ROS and RNS can also cause substantial collateral damage to host tissue [6]. In an appropriately controlled innate immune response, neutrophils additionally contribute to the recruitment of monocytes, macrophages and dendritic cells to the injured site, which in turn activate the adaptive immune responses [6]. Following the recruitment of these immune cells, neutrophils undergo apoptosis and are eventually cleared through subsequent phagocytosis by resident macrophages and dendritic cells [6]. Macrophages, which initially have a pro-inflammatory (M1)-like phenotype can subsequently switch to a resolution-phase macrophage (M2)-like phenotype and begin to rebalance inflammatory homeostasis (Fig. 2) [6].

The resolution phase is triggered when neutrophils become apoptotic and release pre-resolving mediators that contribute to suppressing the continued neutrophil infiltration or macrophage activation [6]. One such mediator is annexin A1, which promotes neutrophil apoptosis and efferocytosis and reduces leukocyte adhesion and migration by interacting with the formyl peptide receptor 2 on the plasma membrane [7]. Another mediator called lactoferrin is known to inhibit the neutrophil migration machinery and produce anti-inflammatory mediators, including IL-10, IL-4 and transforming growth factor (TGF)-β1 [8,9]. Controversially, lactoferrin contributes to extended neutrophil survival in rheumatoid arthritis, an effect dependent on iron status [10]. In the resolution phase macrophages also can secrete several anti-inflammatory cytokines, mainly TGF- β and IL-10 [11].

Numerous studies have expanded our understanding of lipid mediators, illustrating their involvement in promoting the resolution of inflammation [12]. Lipoxins and pro-resolving eicosanoids have been reported to promote the resolution of sterile inflammation. These mediators produced by macrophages via lipoxygenase enzymes facilitate the removal of apoptotic neutrophils and initiate tissue remodeling by enhancing efferocytosis through the macrophage receptor, ALX [11,12]. Lipoxins and protectin D1 from T helper 2 (Th2)-cell have been found to suppress pro-inflammatory cytokines produced by T cells and prevent their infiltration during the resolution phase [12]. Other pro-resolving mediators derived from omega-3 fatty acids and macrophages, such as resolvins, protectins and meresins, can suppress inflammation and tissue damage, leading to restoring homeostasis [13]. In addition, specialized pro-resolving mediators (SPMs) derived from polyunsaturated fatty acid metabolism play an important role in facilitating the transition from the pro-inflammatory phase to the resolution phase of sterile inflammation [14]. On the other hand, leukotriene B4 (LTB4), a pro-inflammatory lipid mediator, has been demonstrated to influence T cell differentiation by favoring the generation of Th17 while reducing the differentiation of regulatory T (Treg) cells [15]. Interestingly, Treg cells boosted efferocytosis through Treg-cell-derived IL-13, which stimulated IL-10 production and promoted the engulfment of apoptotic cells via the guanine nucleotide exchange factor (GEF) Vav1-GTPase Rac1 mechanism [16]. Although IL-13 is typically considered as a pro-inflammatory mediator, it is also demonstrated to be involved in the resolution of inflammation. In murine model of chronic colitis and encephalomyelitis, IL-13 has been shown to induce IL-10 production, suggesting its role in promoting inflammatory resolution [17]. Thus, boosting Treg development has been proposed as an effective therapy for chronic inflammatory diseases [16].

## NLRs and NLRP3 inflammasome

PRRs, expressed on cells of the innate immune system, are a fundamental component in activating the immune response [3]. PRRs play a crucial role in recognizing specific molecular patterns on the surface of pathogens, or in sensing molecules released by damaged host cells. To date, various classes of PRRs have been identified based on factors such as their cellular location and the ligands recognized to induce responses [3]. These include toll-like receptors (TLRs), often found at the cell surface or in endosomes; Nod-like receptors (NLRs) which reside in the cytoplasm; RIG like receptors (RLRs) located in the cytosol and involved in antiviral responses; C type lectin receptors (CLRs), which are the transmembrane receptors with carbohydrate-binding domain; and absence in melanoma 2 (AIM2) like receptors (ALRs) which are associated with a pyrin domain and a DNA binding domain involved in intracellular DNA and DNA damage detection [3,18,19]. Upon binding to these receptors, different signaling pathways such as the nuclear factor κb (NF-κb), mitogen activated protein kinase (MAPK), type I interferon and caspase-1 dependent pathways are activated, leading to further pro-inflammatory responses via upregulation or activation of cytokines and chemokines [3,18,19].

The NLR family is one of the main mammalian PRRs, which plays an essential role in resolving infection and repairing damaged tissue [3]. Structurally, NLRs contain multi-domain proteins that are mainly composed with three parts: leucine-rich repeat (LRR), a receptor domain at C-terminal that can recognize PAMPs or DAMPs; NACHT domain, a central nucleotide-binding domain which is a prerequisite for transducing the following inflammatory signals through formation of the active platforms like inflammasome or endosome; and N-terminal effector domains including a caspase activation and recruitment domain (CARD), a baculovirus inhibitory repeat domain (Bir) or pyrin for binding downstream signalling molecules and could induce inflammatory responses [3]. NLRP3, a crucial intracellular PRRs in antigen-presenting immune cells, is composed of three distinct domains: an N-terminal effector PYD domain, a central NACHT domain and a C-terminal LRR domain. This protein plays a significant role in initiating the formation of inflammasome, which is primarily involved in innate immunity and responsible for regulating caspase-1 dependent inflammatory responses by detecting various stimuli [3,5]. When NLRP3 is activated via PAMPs or DAMPs, ASC, the adaptor protein, is recruited through homotypic PYD–PYD interactions, followed by recruitment of pro-caspase-1 through CARD to assemble an inflammasome. The activation of this complex results in the activation of caspase-1, which contributes to converting pro-IL-1b and pro-IL-18 into their activated and secreted forms, and thereby mediating an inflammatory responses [3,5]. While the exact mechanism remains controversial, there is a two-signal model has been proposed for the signalling pathway of NLRP3 inflammasome activation.

### Priming the NLRP3 inflammasome

**Fig. 3:**
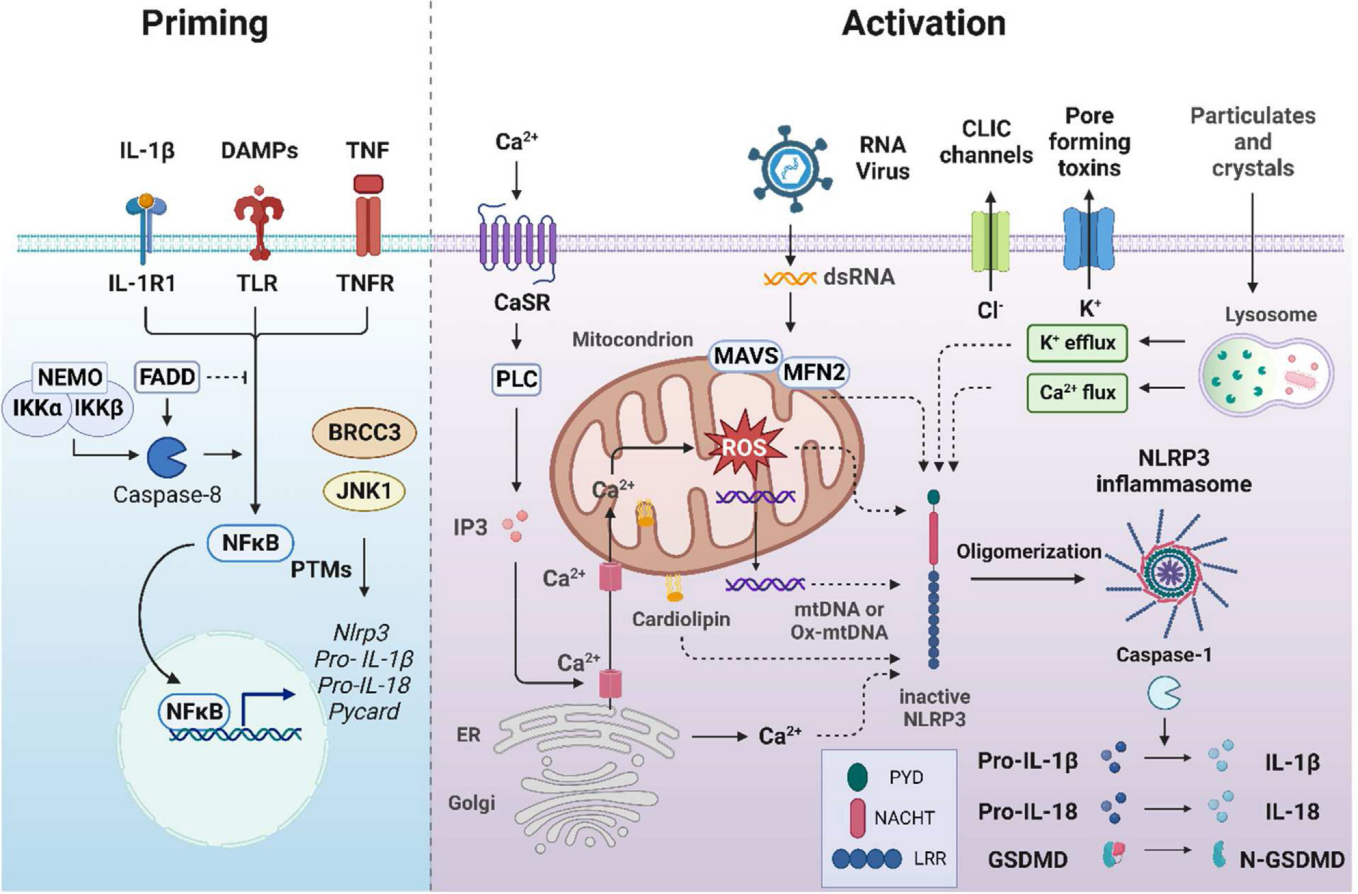
**Two signal model of NLRP3 activation**. There is two-step process involved in activation of NLRP3 inflammasome: Priming (signal 1, left) and Activation (signal 2, right). The priming is mediated by PAMPs/DAMPs recognized by TLRs, leading to the transcriptional upregulation of NLRP3 inflammasome components including NLRP3 itself, pro-IL-1β, Pro-IL-18 and Pycard via an NF-κB-dependent pathway. Caspase-8, FADD and IKK complex are also involved in priming via the regulation of NF-κB activation pathway. Additionally, posttranslational modification can activate NLRP3 inflammasome components by JNK1 and BRCC3. The activation of NLRP3 inflammasome can be triggered by numerous stimuli, such as particulates and crystals, which alter cellular ionic flux and organellar disturbances including ion flux (K^+^, Cl^−^, and Ca^2+^), mitochondrial ROS production, mtDNA, oxidized mitochondrial DNA (Ox-mtDNA) and lysosomal damage. Viral RNA mediates NLRP3 activation via a complex of MAVS and MFN2. Subsequent to NLRP3 inflammasome activation, IL-1β and IL-18 secretion occur.

NLRP3 inflammasome activation requires a priming step that upregulates expression of components such as receptors for DAMPs, TNF and IL-1 receptors, caspase-1 and NLRP3 itself [5]. Caspase-8 and Fas-associated death domain (FADD) have also been implicated in NLRP3 activation during the priming process. Caspase-8 contributes NF-κB transcription and translocation, while FADD plays a dual role in NF-kB signaling and repression of apoptosis [20]. The priming of the NLRP3 inflammasome is also modulated by post-translational modifications [5]. Ubiquitination regulates NLRP3 protein degradation, which is facilitated by E3 ubiquitin ligases F-box L2 (FBXL2), MARCH7, and tripartite motif containing 31 (TRIM31) [21,22]. Conversely, deubiquitinating enzymes like BRCC3 maintains NLRP3 activity by cleaving Ly63-linked polyubiquitin chains, suggesting E3 ligases or deubiquitinating enzymes as targetable proteins for immune modulation [23]. Phosphorylation mediated by kinases such as Syk, Jnk and Bruton's tyrosine kinase have been shown to facilitate NLRP3 activation [24]. In conclusion, the priming of the NLRP3 inflammasome requires both the upregulation of NLRP3-related gene expression and the involvement of post-translational modifications, highlighting many potential avenues for therapeutic intervention in the modulation of inflammatory pathogenesis. Subsequently, the activation step leads to functional outcomes dependent on NLRP3 [5] (Fig. 3).

### Activating the NLRP3 inflammasome

Following a priming step, the NLRP3 inflammasome can be activated by various cellular events including ion flux (such as K^+^ efflux or Ca^2+^ mobilization), mitochondrial dysfunction, and lysosomal damage which induce signaling pathways leading to NLRP3 oligomerization [25] (Fig. 3).

K^+^ efflux, observed in response to various intracellular stimuli, is well established to activate the NLRP3 inflammasome [26]. Both depleted cytosolic K^+^ and high extracellular K^+^ levels triggers NLRP3-mediated inflammation, mediated by IL-1β maturation and caspase-11 activation [26,27]. Reduced intracellular K^+^ levels are positively correlated with IL-1β secretion in macrophages, and this is facilitated by Ca^2+^ independent phospholipase A2 [26,27].

Elevated extracellular Ca^2+^ also triggers NLRP3 inflammasome activation, while chelating Ca^2+^ inhibits IL-1β secretion, underscoring the importance of Ca^2+^ in the activation process [28] The Ca^2+^-sensing receptor (CaSR) activates the NLRP3 inflammasome by increasing intracellular Ca^2+^ and decreasing cyclic AMP (cAMP), which involves the activation of phospholipase C and subsequent release of Ca^2+^ from ER [29]. Mitochondrial oxidative stress, induced by Ca^2+^ overload, causes mitochondrial dysfunction, accumulation of ROS, and the release of oxidized mitochondrial DNA (mtDNA) into the cytosol, further contributing to the activation of NLRP3 [30].

Upon stimulation, NLRP3 relocates from the ER to the perinuclear space, where it interacts with mitochondria and mitochondria-associated membranes (MAMs) via proteins such as cardiolipins and mitofusin2 (MFN2) [31]. Cardiolipin is primarily localized in the inner mitochondrial membrane but can be exposed to the outer membrane during mitochondrial stress, where it binds directly to the NLRP3 inflammasome [31]. MFN2 forms a complex with mitochondrial antiviral signaling protein (MAVS) to promote the recruitment of NLRP3 to mitochondria and subsequently contribute to IL-1β secretion [32,33].

Lysosomal disruption triggers NLRP3 inflammasome by releasing particulates into the cytosol [34]. Studies have shown that phagocytosis of cholesterol crystals, silica, or lysosomotropic dipeptide L-leucyl-l-leucine methyl ester (LLOMe) induced lysosomal rupture, activating NLRP3 inflammasome [34]. Lysosomal cysteine cathepsins, the major proteases in the lysosome, also contribute to NLRP3 inflammasome activation since inhibition of cathepsins has been shown to partially limit NLRP3 activation [35].

## NLRs in cardiometabolic diseases

**Fig. 4:**
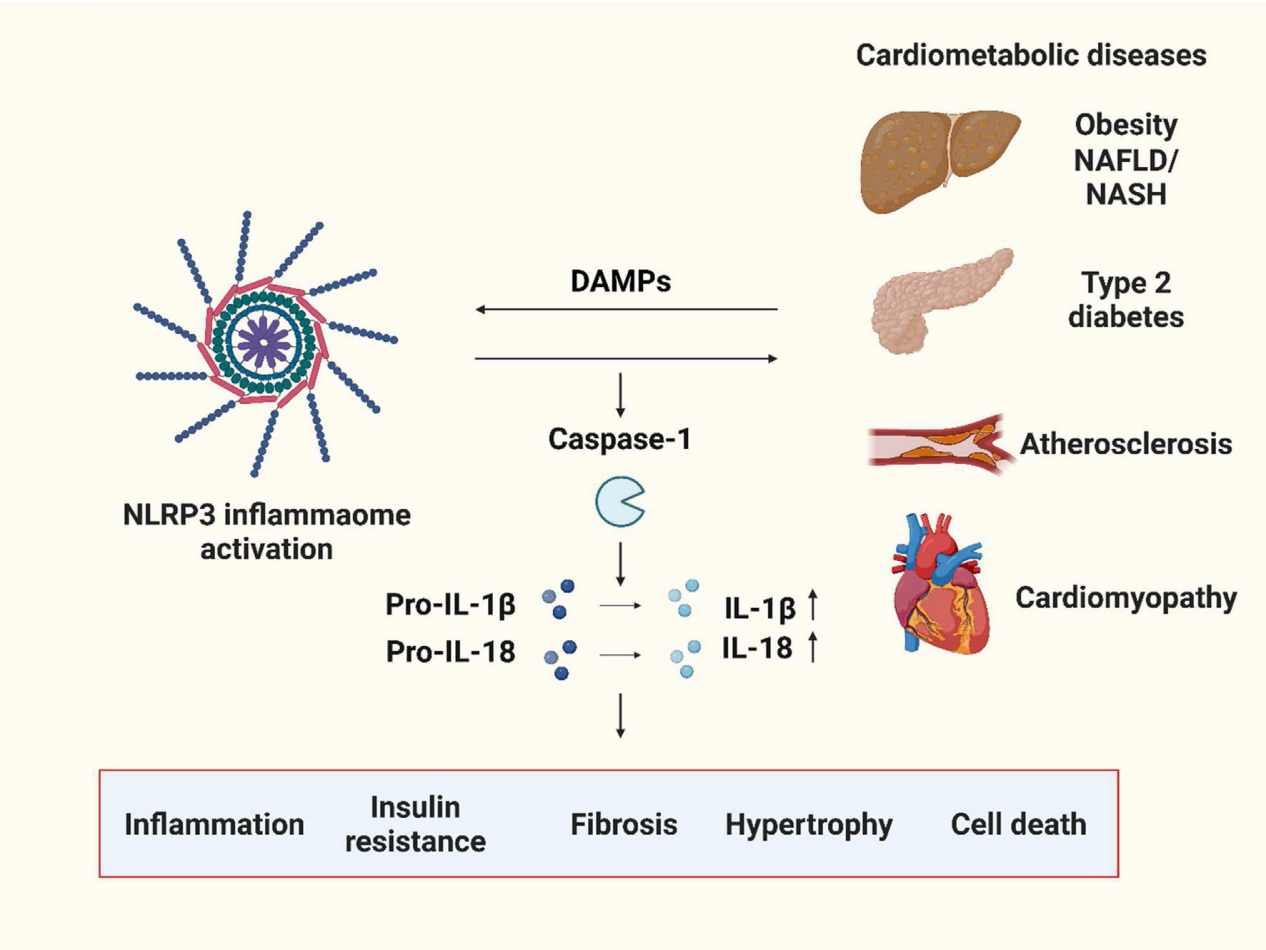
**NLRP3 inflammasome and cardiometabolic diseases**. The excess activation of NLRP3 inflammasome, triggered by DAMPs and other inducers from various cell types, further contributes to cardiometabolic disease pathogenesis via altering inflammation, insulin resistance, fibrosis, hypertrophy and cardiac cell death.

As the NLRs are a family of modular cytosolic sensors responding to DAMPs and PAMPs, several members of NLR family have been shown to be involved in the development of chronic inflammation-related diseases. In Table 1 we summarize current knowledge from studies on the role of NLRs in various cardiometabolic diseases. NLRP3 is the most well-studied inflammasome and a strong association with cardiometabolic diseases has been established, thus making it an attractive therapeutic target (Fig. 4).

**Table 1:** Studies implicating NLRs in cardiometabolic diseases.

Category	Specific disorder (disease)	Models, Cells or Tissue studied	NLRs studied	Observations	Reference
Metabolic diseases	Metabolic syndrome	Subcutaneous adipose tissue and serum from MetS patients	NOD1, NOD2	Increased expression for NOD1 in adipose tissue with increased circulating MCP-1, IL-6 and IL-8, whereas no significant changes in NOD2 expression.	[36]
		Subcutaneous and visceral adipose tissue-derived stem cells in MetS patients	NLRP3	Increased NLRP3 inflammasome activation, TGF-β1 secretion and suppressed lymphocyte proliferation and macrophage M2 polarization	[37]
		White adipose tissue, *Nod1*^−/−^ mice	NOD1	NOD1 expressed in adipocytes, and mediates adipocyte lipolysis.	[38]
	Obesity and/or Type 2 Diabetes (T2D)	Liver and adipose tissue in high fat diet (HFD)-fed *Nod2*^−/−^ mice	NOD2	Enhanced HFD-induced metabolic inflammation and insulin resistance in *Nod2*^−/−^ mice	[39,40]
		Subcutaneous and visceral adipose tissue from cohorts with metabolic dysfunction	NLRP3	Increased expression of NLRP3, IL-1β, and increased number of macrophages with higher casepase-1 activity	[41]
		Abdominal subcutaneous adipose tissue from obese participants, and *Nlpr3*^−/−^ mice	NLRP3	Increased expressions of Nlpr3, ASC, IL-1β and activity of caspase-1	[42]
		HFD-fed, *Nod1*^−/−^ mice	NOD1	NOD1 level was elevated by HFD feeding, and deficiency of NOD1 protected against HFD-induced glucose and insulin intolerance.	[43]
		HFD-fed *Nlrp*1^−/−^, *Nlrp1*Mut, *Il18*^−/−^, *Il1r*^−/−^ mice	NLRP1	Loss of Nlrp1 exacerbated obesity. Increased Nlrp1 expression attenuated HFD-induced metabolic syndrome via IL-18-mediated lipolysis	[44]
		HFD-fed *Nlrp3*^−/−^ mice	NLRP3	*Nlrp3*^−/−^ prevented LV concentric remodelling and metabolic stress	[45]
		HFD-fed mice	NOD1	A positive correlation between the level of transcripts for NOD1 and inflammation in metabolic organs.	[46]
		HFD-fed, *Nod2*^−/−^ mice	NOD2	NOD2 deletion caused increased M1 macrophage polarization	[47]
		Subcutaneous abdominal adipose tissue from obese patients	NOD1, NOD2	Increased expressions of NOD1, NOD2 and proinflammatory cytokines including MCP-1, IL-6 and IL-8	[48]
		HFD-fed, *Nod1/2*^−/−^ mice	NOD1, NOD2	Deletion of both NOD1 and NOD2 was protective against HFD-induced inflammation, lipid accumulation and insulin intolerance.	[49]
		HFD-fed, *Fat-1* transgenic mice	NLRP3	Increased HFD-induced expression of NLRP3, production of IL-1β and IL-18, and increased activity of caspase-1 in adipose tissue	[50]
		HFD-fed, or STZ-induced diabetic Sprague–Dawley rats	NLRP3	Increased expression of NLRP3 inflammasome components (NLRP3, ASC, caspase-1, IL-1β), NF-kB, and Nrf2 in liver	[51]
	T1D	Blood mononuclear cells (PBMCs) from T1DM patients	NLRP1, NLRP3	Lower NLRP1, NLRP3, caspase-1 and IL-1β expressions.	[52]
		STZ-induced T1DM rat	NLRP3	Increased NLRP3, IL-18, IL1β and GSDM-D protein levels in nucleus pulposus tissues	[53]
	NAFLD	Liver and primary hepatocyte from HFD-fed mice	NLRP3	Increased expressions of NLRP3, ASC, caspase-1, and increased caspase-1 activity and IL-1β level	[54]
		Visceral adipose tissue from NAFLD/NASH patients	NLRP4, NLRP6	Downregulated NLRP4, IL-1β, and upregulated NLRP6 expression in visceral adipose tissue, and increased circulating IL-18 levels in patients with pericellular fibrosis.	[55]
		Liver samples from NAFLD patients, and *ob/ob* or *Nlrp2*^−/−^ mice	NLRP2	Decreased mRNA and protein expression of Nlrp2 in NAFLD patient, and *Nlrp2*^−/−^ enhanced HFD-induced metabolic syndrome and insulin resistance.	[56]
	Gestational diabetes	Subcutaneous and omental adipose tissue from gestational diabetes mellitus (GDM) women.	NOD1	Expression of NOD1 significantly higher in GDM women than normal glucose tolerant women.	[57]
Heart failure					
CVD	Atherosclerosis	Carotid atherosclerotic plaques and serum from patients	NLRP3	Increased expression of NLRP3 signalling pathway components (NLRP3, ASC, IL-1β, and IL-18)	[58]
		Aorta of FK565-induced atherosclerosis model of *ApoE*^−/−^ mice and *ApoE*^−/−^*Nod1*^−/−^ mice	NOD1	Required for atherosclerosis development	[59]
		Atherosclerotic plaques from myocardial infarction patients	NLRP3	Increased expression of NLRP3 signalling components (NLRP3, ASC, CASP1, IL-1β, and IL-18)	[60]
		*ApoE*^−/−^, *CD36*^−/−^ mice	NLRP3	Increased NLRP3 inflammasome priming, activation and IL-1β production	[61]
		*LdlR*^−/−^ diabetes mouse models with bone marrow transplantation from *Nlrp3*^−/−^ donor	NLRP3	Increased circulating levels of IL-1β and IL-18 in diabetic mice, and reduced atherosclerotic lesions in mice with hematopoietic deletion of NLRP3	[62]
	Metabolic cardiomyopathy	HFD-fed *NLRP3*^−/−^, *ASC*^−/−^ mice	NLRP3	No change in cardiac hypertrophy, but attenuated obesity-induced LV concentric remodelling and systemic metabolic dysregulation in *NLRP3*^−/−^ and *ASC*^−/−^ mice	[63]
		HFD-fed mice with TAC surgery	NLRP3	Attenuated adverse cardiac remodelling with improved cardiac metabolism by NLRP3 inhibition	[64]
		*Nlrp3*^−/−^, Calcineurin-transgenic (CNTg) mice	NLRP3	Increased IL-1β secretion and Nlrp3 gene expression in hypertrophic heart	[65]
		Diabetic cardiomyopathy rat model with HFD/streptozotocin	NLRP3	Increased activation of NLRP3, caspase-1 and IL-1β	[66]
		High fructose diet-fed mice	NLRP4	NLRP4 attenuates high fructose-induced cardiac injury, and the secretion of Tgf-β1, Tnf-α, IL-1β IL-18 and IL-6 via reducing TBK1-IRF3 and IKK/NF-kB signaling pathways	[67]
	Hypertrophy	*Nlrp3*^−/−^, *P2rx7*^−/−^, adrenergic neuron specific *SLC174A9*^−/−^ mice	NLRP3	Suppressed pressure overload-induced cardiac dysfunction in *Nlrp3*^−/−^ mice	[68]
		Left ventricle samples from HF patients and *Nlrp3*^−/−^ mice with TAC surgery	NLRP3	Downregulated NLRP3 expression in failing heart	[69]
		TAC-induced hypertrophy mice model	NLRP3	Upregulated NLRP3, IL-1β, cleaved caspase-1, and GSDMD-N	[70]
		*Nlrp1*^−/−^ mice with aortic banding surgery	NLRP1	Nlrp1^−/−^ protects against cardiomyocyte hypertrophy with reduced MAPK signalling pathways.	[71]
	Ischemia (I) ± reperfusion (R) heart injury	*ASC*^−/−^ mice, *Caspase 1*^−/−^ mice with I/R	NLRP3	Reduced infarcted area, reduced inflammatory cell infiltration, and improved cardiac remodelling	[72]
		*NLRP3*^−/−^ mice, *ASC*^−/−^ mice with I/R	NLRP3	larger infarct size 24 h after I/R	[73]
		I mouse model	NLRP1	Increased NLRP1 activation and autophagic flux promotion suppress NLRP1 inflammation in myocardial infarction	[74]
		I mouse model	NLRP3	Increased expression of NLRP3 inflammasome, IL-1β and IL-18	[75]
		I/R mouse model	NLRP3	Increased myocardial NLRP3 expression	[76]
		*NLRX1*^−/−^ mice with I/R	NLRX1	Increased infarct size, enhanced lactate production and glucose oxidation relative to fatty acid oxidation	[77]

### NLRP3 in obesity, diabetes and nonalcoholic fatty liver disease

Obese patients exhibit fundamental changes in cellular metabolism and are characterized by chronic low-grade inflammation, particularly in adipose tissue [78]. In obese patients as well as diabetic mouse models, upregulation of NLRP3 inflammasome components and overexpression of TNF-α were found in adipose tissue [37,41]. Underlying mechanisms include release of DAMPs from necrotic adipocytes [78] or high lipopolysaccharide (LPS) levels in circulation as a result of altered microbial composition and impaired gut permeability [63]. Importantly, *Nlrp3*^*−/−*^ mice were prevented from HFD-induced metabolic dysfunctions [42]. HFD-fed *Nlrp3*^*−/−*^ mice were also observed to have reduced adipocyte size, reduced macrophage infiltration and suppressed inflammation in fat depots and liver, which contributed to improved insulin sensitivity [42]. Similar evidence has been shown in mice lacking IL-1 receptor I (*IL-1RI*) where there was less inflammation in adipose tissue, and improved glucose homeostasis after HFD treatment [78]. Likewise, pharmacological inhibition of NLRP3 inflammasome can also prevent HFD-induced non-alcoholic fatty liver disease with improved insulin sensitivity, and reduced liver damage accompanied by decreased caspase-1 activity and IL-1β expression levels versus wild-type mice [78]. However, a contradicting study indicated that there was no prevention effect of *Nlrp3*^*−/−*^ in mice against HFD-induced metabolic disturbances and inflammation [79]. Several reasons could affect the discrepancies between these observations, including environmental factors, such as diet composition, feeding duration and microbiota profile. As metabolic complications are associated with aging, the regulation of NLRP3 inflammasome may also mediate age-related chronic inflammation. A study suggested that NLRP3-mediated IL-1β and IL-18 expression in aged mice impaired metabolic homeostasis [80].

Of note, the NLRP3 inflammasome was also reported to be involved in development of statin-induced insulin insensitivity. Statins are lipid-lowering medications that are commonly prescribed for prevention and treatment of cardiovascular evets, yet they have been shown to significantly increase the risk of new onset diabetes via increased NLRP3 activation [81,82]. Henriksbo and colleagues demonstrated that mice treated with statins exhibited insulin resistance in adipose tissue with concomitant activation of the NLRP3/caspase-1 axis [83,84]. Treatment of LPS-primed 3T3-L1 adipocytes with statins activated NLRP3 and impaired insulin signaling, the latter not being dependent on IL-1β secretion. They identified a mechanism of statin-induced reduction in prenylation isoprenoids leading to NLRP3 activation and insulin resistance. Subsequent studies have indicated that statin-mediated activation of the NLRP3 inflammasome preceded insulin resistance and occurred via p38 and mTOR signaling [85].

Altogether, existing literature clearly provides strong rationale for targeting NLRP3-induced inflammation as a therapeutic approach across a span of cardiometabolic diseases.

## NLRP3 in atherosclerosis

Atherosclerosis is a chronic inflammatory disease resulting from the formation of atherosclerotic plaques in the arterial wall in which extensive immune cell infiltration and vascular smooth muscle cell proliferation occur [59]. An association between NLRP3 and atherosclerosis pathogenesis has been suggested based on evidence such as a large amount of NLRP3 inflammasome-related components, such as NLRP3, ASC, caspase-1, IL1-β and IL-18 detected in atherosclerotic plaques [59]. Notably, a strong and positive correlation between NLRP3 and the severity of coronary artery stenosis is further observed in patients with atherosclerosis [62].

In preclinical studies, the direct function of NLRP3 on atherosclerosis pathology has been explored. Results indicated that atherosclerotic progression was attenuated by NLRP3 inhibition and IL-1β production [86]. Mechanistically, the major triggers for NLRP3 inflammasome are oxidized LDL (oxLDL), cholesterol crystals and calcium phosphate crystals in atherosclerosis. The oxLDL induces inflammation by cholesterol crystallization, NLRP3 priming/activation and pro-IL-1β expression, while in CD36 deficient macrophages, oxLDL failed to release IL-1β [62]. Of note, excessive cholesterol crystals can cause lysosomal destabilization and rupture, resulting in leakage of cathepsin B and activation of NLRP3 inflammasome [5]. A study demonstrated that the cholesterol crystals induced acute inflammation, which was impaired in mice deficient in NLRP3 inflammasome components [79]. Such results additionally reflect the role of crystalline cholesterol as a DAMP in NLRP3 inflammasome activation. One recent study consistently demonstrated that bone marrow transplantation from mice deficient in *caspase 1/11* to *Ldlr*^−/−^ mice resulted in significantly reduced atherosclerotic plaques, and decreased inflammasome-dependent IL-18 levels after atherogenic diet feeding for 8 weeks when compared to the control bone marrow [88]. On the contrary, another study illustrated that there was no relationship between NLRP3 inflammasome and atherosclerosis pathogenesis by using *ApoE*^*−/−*^ *Nlrp3*^*−/−*^, *ApoE*^*−/−*^ *Asc*^*−/−*^ and *ApoE*^*−/−*^ *caspase-1*^*−/−*^ double-deficient mice [89]. In summary, most clinical and preclinical studies provide evidence for the important role of NLRP3 inflammasome in atherosclerosis.

## NLRP3 in cardiomyopathy

Cardiomyopathy is associated with systemic metabolic disorders across a broad spectrum of pathological foundations, including obesity, diabetes and alcoholism [90]. This type of heart failure in the absence of any ischemic injury or hypertension is characterized by structural and functional alterations including interstitial fibrosis [90]. In cases of obesity, chronic low-grade inflammation leads to myocardial cellular abnormalities including mitochondrial dysfunction, endoplasmic reticulum stress, and dysregulated calcium handling, and eventually results in dysfunctional myocardial relaxation [6]. Collectively, the progressed inflammation state in the heart showed increased immune cell infiltration, neurohormonal activation, cardiomyocyte death, fibrosis and compromised diastolic and/or systolic function.

Numerous studies have demonstrated that cardiometabolic patients experience myocardial remodeling, which involves systemic inflammation. This inflammation is triggered by inflammatory cytokines, circulating metabolic substrates and dysregulated immune processes [69]. In particular, in cardiometabolic patients, there is a persistent myocardial inflammation caused by the activation of innate immune cells through PRRs like TLR4 and NLRP3 inflammation, which activate NF-κB-dependent pathways [69]. In addition, the activation of NLRP3 inflammasome/caspase-1 was also observed in several mouse models of metabolic cardiomyopathy. After 52 weeks HFD feeding, mice showed left ventricle (LV) concentric remodelling and impaired diastolic function with severe cardiac fibrosis and inflammation, a phenotype that was attenuated in *Nlrp3*^*−/−*^ or *Asc*^*−/−*^(*Pycard*^*−/−*^) mice [50]. Another study illustrated that administration of MCC950, a specific NLRP3 inhibitor, attenuated adverse cardiac remodelling and improved cardiometabolic dysfunction induced by HFD feeding and transverse aortic constriction surgery in mice [64]. Cardiomyocyte-specific expression of the constitutively active calcineurin transgene (CNTg) in mice showed hypertrophic cardiomyopathy with cardiac inflammation [65]. When *NLRP3* is genetically ablated in CNTg mice, IL-1β maturation was reduced, which ameliorated cardiac inflammation with improved systolic performance [65]. Moreover, T2D mice were observed with increased sterile inflammation via upregulated TLR2 and NLRP3 inflammasome-mediated IL-1β production in myocardial macrophages, which led to cardiac arrhythmias. However, diabetic mice with *Nlrp3* and *Caspase-1* deletion showed lower vulnerability and reduced severity of cardiac arrhythmia [91]. By pharmacological intervention with IL-1βR antagonist or NLRP3 inhibitor, cardiac arrhythmias were ameliorated, which indicated that activation of NLRP3 inflammasome was implicated in diabetic cardiomyopathy [91]. Finally, in another study knockdown of *Nlrp3* inhibited the formation of active IL-1β and IL-18, which also prevented diastolic LV dysfunction in diabetic rats [91]. Collectively, these findings suggest that NLRP3 inflammasome-induced sterile inflammation plays a vital role in the development of cardiac dysfunction.

## NLRP3 in cardiac hypertrophy

Cardiac hypertrophy is an adaptive remodeling response of the heart to pathological stress, which can result from various factors such as pressure overload, volumetric stress, mutations of sarcomeric proteins, or loss of contractile mass [92]. In hypertrophic models, NLRP3-mediated inflammation has been implicated in driving adverse cardiac remodeling [92]. Cardiac hypertrophy was induced in mice through a transverse aortic constriction (TAC) and this led to a significant increase in NLRP3 inflammasome components (NLRP3, ASC, and IL-1β). Furthermore, expression of genes such as *Nppa*, *Myh7, Col1a1, Vegfa, Il6*, *Mcp1*, and *Tnfα* increased in myocardial tissue, suggesting adverse cardiac remodeling [68]. However, these changes were suppressed in hearts from *Nlrp3*^*−/−*^ mice versus wild type [68]. Interestingly, this study also indicated that cardiac inflammation and hypertrophy are regulated by heart–brain interaction, providing an example of crosstalk between myocardial and neural signals in cardiac hypertrophy [68].

In addition to genetic disruption of NLRP3, pharmacological intervention inhibiting NLRP3 attenuated TAC-induced cardiac hypertrophy in mice [70]. Hypertrophic mouse hearts induced by TAC showed significantly increased levels of NLRP3, ASC, cleaved caspase-1, and GSDMD-N proteins, along with compromised cardiac function. However, this was corrected by administering irisin [4]. Nevertheless, another study suggested that NLRP3 protein levels were significantly decreased in the heart of mice 1–6 weeks after TAC, as well as in heart biopsy samples from patients with dilated cardiomyopathy [69]. Cardiomyocyte hypertrophy induced by TAC was exacerbated in *Nlrp3*^*−/−*^, but it was corrected by TLR4 antagonist eritoran, leading to improved cardiac fibrosis and inflammation [69].

Therefore, despite extensive research, further understanding is needed to determine the exact role of NLRP3 and confirm when and via which mechanism it is beneficial or detrimental in cardiac remodeling during hypertrophy.

## NLRP3 in ischemia heart diseases

Myocardial ischemia is a typical consequence of coronary atherosclerosis which could lead to irreversible injury upon severe and sustained ischemia [69]. Although early reperfusion is the first choice to use in patients with acute myocardial infarction, reperfusion injury occurs leading to inflammation that can eventually contribute to the development of heart failure. In 2011, Kawaguchi et al. demonstrated that cardiac biopsy tissues from patients with myocardial infarction were infiltrated with a large number of inflammatory macrophages and neutrophils and high expression of ASC [69]. Consistent with this finding, mice with ASC or caspase 1 deficiency are also protected against ischemia/reperfusion (I/R) injury associated with reduced infarcted area, reduced inflammatory cell infiltration, and improved cardiac remodeling [69]. In addition, the expression of NLRP3 inflammasome, and it is downstream inflammatory cytokines IL-1β, and IL-18 was markedly increased in the left ventricle after myocardial infarction in both an animal model of ischemia induced by ligation of the coronary artery and in patients with coronary artery disease [75,93]. Moreover, Toldo et al. demonstrated that NLRP3 myocardial expression was increased after long-term reperfusion time (6hrs and 24hrs). The administration of NLRP3 inhibitor given 1hr after reperfusion failed to aggravate infarct size with reduced caspase-1 activity measured at 24hr, indicating NLRP3 inflammasome activation aggravates I/R injury whereas inhibition of it significantly alleviates the I/R damage by reducing infarct size [76]. However, contradictory results were reported in a study showing a larger infarct size in both *Nlrp3*^*−/−*^ mice and *Asc*^*−/−*^ mice 24 h after I/R. Such discrepancy might be due to the different time points chosen for assessment since the inflammasome was not activated at the early stage of myocardial infarction (MI) [73].

Caspase-1 also displayed a detrimental role in an MI model by promoting pyroptosis [94]. Results showed that diabetic rats exhibited a larger myocardial infarct size, higher Creatine Kinase MB (CK-MB) and lactate dehydrogenase (LDH) release after myocardial I/R, which was accompanied with increased NLRP3 inflammasome activation [94]. Additional evidence showed that NLRP3 inflammasome activation occurred in fibroblasts and endothelial cells after I/R. Interestingly, it was observed using an I/R injury model that application of thioredoxin-interacting/inhibiting protein (TXINP) siRNA diminished the formation and activation of the NLRP3 inflammasome in endothelial cells, but not cardiomyocytes [95].

Overall, the majority of published studies support the notion that NLRP3 inflammasome contributes significantly to the development of ischemic heart injury, and thus represents a viable therapeutic target.

## IL-1β therapeutics in cardiometabolic disease

IL-1 is a classic proinflammatory cytokine that exists in two isoforms: IL-1α and IL-1β, which can function as autocrine, paracrine, and endocrine messengers [96]. IL-1β is a principal cytokine depending on activation via cleavage by caspase-1 after NLRP3 inflammasome activation. Elevated IL-1β activity contributes to the pathogenesis of conditions such as insulin resistance, adverse cardiac remodeling, and heart failure and it thus emerged as a therapeutic target, especially for inflammatory, metabolic, and cardiovascular diseases [96,97].

The recent success of canakinumab, a humanized monoclonal antibody that neutralizes IL-1β, has drawn attention to and accelerated further development of drugs targeting IL-1β and IL-1 receptor. Canakinumab demonstrated potent anti-inflammatory effects and has received clinical approval for treating autoinflammatory diseases, including rheumatoid arthritis and cryopin-associated autoinflammatory syndromes (CAPS) [98]. In the Canakinumab Anti-inflammatory Thrombosis Outcome Study (CANTOS) trial, patients with atherosclerotic disease who received canakinumab exhibited significantly lower levels of inflammation, as indicated by reduced systemic C-reactive protein and IL-6, independent of lipid-level lowering. Additionally, these patients experienced a significantly lower incidence of recurrent cardiovascular events compared to the placebo group [98]. Anakinra, a recombinant human IL-1 receptor antagonist, has also demonstrated improved clinical outcomes in patients with acute myocardial infarction [99]. Specifically, patients who received anakinra showed a significant reduction in C-reactive protein, leukocyte counts, and neutrophils and an increase in eosinophils, indicating the alleviation of inflammation [99]. Another recombinant IL-1 blocker, rilonacept, which functions as an IL-1α and IL-1β trap, has shown beneficial effects on patients with recurrent pericarditis in the phase 3 clinical trial, RHAPSODY [100]. Patients who received rilonacept experienced a rapid resolution of recurrent pericarditis episodes than placebo [100]. Therefore, drugs targeting IL-1β have demonstrated improved clinical outcomes in cardiovascular disease, including atherosclerosis, myocardial infarction and pericarditis.

## Conclusions

As the number of patients with cardiometabolic diseases rises due to factors such as Western diet, a sedentary lifestyle and an aging population, the demand for effective therapeutics rises. With current advances in understanding the role of NLRP3 in various cardiometabolic diseases, targeting NLRP3 itself or related components as a therapeutic for cardiometabolic diseases has gained considerable interest. Current therapeutic approaches related to NLRP3 have mainly focused on the inhibition of IL-1β, and these have been highly successful. Therefore, it is also of interest to consider the advantages, or disadvantages, of specifically targeting NLRP3 itself. Persevering with mechanistic studies that will enable the refinement of strategies and scenarios to target NLRP3 will help future clinical translation, with the potential for integrating precision medicine in inflammation-related cardiometabolic diseases.

## Conflicts of interest

The authors have no conflicts of interest to declare.
